# Emergence of Vaccine-Derived Polioviruses during Ebola Virus Disease Outbreak, Guinea, 2014–2015

**DOI:** 10.3201/eid2401.171174

**Published:** 2018-01

**Authors:** Maria Dolores Fernandez-Garcia, Manasi Majumdar, Ousmane Kebe, Aichatou D. Fall, Moussa Kone, Mouctar Kande, Moustapha Dabo, Mohamed Salif Sylla, Djenou Sompare, Wayne Howard, Ousmane Faye, Javier Martin, Kader Ndiaye

**Affiliations:** Institut Pasteur, Dakar, Senegal (M.D. Fernandez-Garcia, O. Kebe, A.D. Fall, O. Faye, K. Ndiaye);; National Institute for Biological Standards and Control, Potters Bar, UK (M. Majumdar, J. Martin);; World Health Organization, Conakry, Guinea (M. Kone, M. Kande);; Guinean Ministry of Health, Conakry (M. Dabo, M.S. Sylla, D. Sompare);; National Institute for Communicable Diseases, Johannesburg, South Africa (W. Howard)

**Keywords:** vaccine-derived poliovirus, Ebola outbreak, acute flaccid paralysis, viruses, Guinea, vaccines, vaccination

## Abstract

During the 2014–2015 outbreak of Ebola virus disease in Guinea, 13 type 2 circulating vaccine-derived polioviruses (cVDPVs) were isolated from 6 polio patients and 7 healthy contacts. To clarify the genetic properties of cVDPVs and their emergence, we combined epidemiologic and virologic data for polio cases in Guinea. Deviation of public health resources to the Ebola outbreak disrupted polio vaccination programs and surveillance activities, which fueled the spread of neurovirulent VDPVs in an area of low vaccination coverage and immunity. Genetic properties of cVDPVs were consistent with their capacity to cause paralytic disease in humans and capacity for sustained person-to-person transmission. Circulation ceased when coverage of oral polio vaccine increased. A polio outbreak in the context of the Ebola virus disease outbreak highlights the need to consider risks for polio emergence and spread during complex emergencies and urges awareness of the challenges in polio surveillance, vaccination, and diagnosis.

Poliovirus, the etiologic agent of paralytic poliomyelitis, can cause acute flaccid paralysis (AFP) (*1*). Three serotypes of poliovirus (1, 2, and 3) belong to *Enterovirus species C* (family *Picornaviridae*, genus *Enterovirus*). Since the World Health Organization (WHO) and partners launched the Global Polio Eradication Initiative in 1988, the widespread use of live-attenuated oral poliovirus vaccine (OPV) has been crucial for reducing polio cases >99% (*2*). Although OPV has many advantages (easy administration by mouth, low cost, effective intestinal immunity, and durable humoral immunity), it has the disadvantage of genetic instability. Because of the plasticity and rapid evolution of poliovirus genomes and selective pressures during replication in the human intestine, vaccine poliovirus can lose key genetic determinants of attenuation through mutation or recombination with closely related polio and nonpolio enterovirus strains, acquiring the neurovirulence and infectivity characteristics of wild-type poliovirus (WPV) (*3*). Because of this genetic instability, in settings where a substantial proportion of the population is susceptible to poliovirus, OPV use can lead to poliovirus emergence and sustained person-to-person transmission and spread in the community of genetically divergent circulating vaccine-derived polioviruses (cVDPVs). cVDPVs (as well as VDPVs excreted by immunodeficient persons) are defined as those with >1% nt sequence divergence (for polioviruses types 1 and 3) or >0.6% (for poliovirus type 2) in the major viral capsid protein coding region 1 (VP1) of the corresponding OPV strain (*3*).

The first known outbreak of cVDPV infection was reported in Hispaniola in 2000–2001 (*4*), although retrospective analyses of poliovirus isolates from Egypt during the 1960s suggest that this phenomenon might have been more common than anticipated. Although cVDPV outbreaks have been associated with all 3 components of trivalent OPV, most (97%) VDPV isolates that have emerged from OPV use are type 2 (*5*). Since 2005, type 2 cVDPV outbreaks have occurred in 17 countries of Africa and Asia, causing ≈600 cases of paralytic polio (*5*–*7*).

In Guinea, AFP case surveillance was established in 1997 as part of the Global Polio Eradication Initiative; fecal samples were tested at the WHO Reference Polio Laboratory in Senegal. AFP surveillance and vaccination with OPV led to the interruption of WPV transmission in Guinea, where the last known indigenous WPV type 1 dates back to 2009, and the last case of wild-type paralytic poliomyelitis was detected in August 2011 after importation of a type 3 WPV from Côte d’Ivoire.

We report the emergence and spread of type 2 cVDPVs in Guinea in 2014–2015 during the Ebola virus disease (EVD) epidemic. In 2014, WHO declared that EVD and polio were Public Health Emergencies of International Concern (PHEICs). We describe the occurrence of a PHEIC within a PHEIC. The occurrence of a polio outbreak in the context of the Ebola emergency highlights the need to evaluate the challenges in polio surveillance, vaccination, and diagnosis during complex emergencies to improve prevention and response strategies for future outbreaks in high-risk areas. To clarify the genetic properties of cVDPVs and to reconstruct the events leading to their emergence and spread, as well as the subsequent public health responses used to stop the polio epidemic, we analyzed epidemiologic and virologic data of polio cases in Guinea.

## Materials and Methods

### Surveillance of AFP Cases

We identified patients with paralytic poliomyelitis through the Guinea AFP surveillance system according to WHO guidelines (*8*). Through this system, district health officers routinely investigate AFP cases reported by health centers and hospitals, collect fecal samples, investigate clinical histories, record the total number of OPV doses received, and assess residual paralysis 60 days after onset. During September 2015–December 2016, additional fecal samples were collected from contacts of most AFP patients. According to the outcome of laboratory investigations, AFP cases were classified as confirmed polio or discarded as non–polio-associated AFP (*8*).

### Epidemiologic Outbreak Investigation

To assess epidemiologic factors associated with the outbreak, during December 17–28, 2015, we conducted field investigations in Siguiri and Kankan Prefectures, Guinea. The investigation team was composed of trained healthcare workers from the Ministry of Health, WHO, and UNICEF. The workers interviewed parents/caregivers of 5 children from whom laboratory-confirmed type 2 cVDPV had been isolated (3 case-patients [nos. 15-078, 15-114, and 15-115] and 2 contacts [15-115-C4, 15-115-C5]) and parents/caregivers of potential contacts in the community. Data were collected through face-to-face interviews with the parents/caregivers by use of a structured questionnaire. The specific questions included vaccination history (routine and supplementary), travel history, household demographics, living conditions, water supply, and sanitary conditions. Vaccination status was checked from immunization cards (where available) or by a convincing history of vaccination from the parent/caregiver. Finally, the team conducted active searches in the communities for new AFP cases, collected fecal samples from contacts, raised awareness in families to promote poliomyelitis vaccination, and assessed 5 health centers in both prefectures.

### Assessment of Vaccination Coverage

Supplementary immunization activities (SIAs) are mass vaccination campaigns conducted in a short period, during which a dose of OPV is administered to all children <5 years of age, regardless of previous vaccination history (*9*). Data on routine vaccination coverage with 3 doses of OPV (OPV3) were available from administrative reports of routine vaccination.

### Virus Isolation and Molecular Typing

Polioviruses were isolated from fecal samples according to WHO standard procedures (*10*) and subjected to intratypic differentiation by reverse transcription PCR targeting the VP1 region, according to Centers for Disease Control and Prevention protocol (*11*). During 2013–2015, typing of nonpolio enterovirus isolates was performed as previously described (*12*).

### Sequencing and Sequence Analysis

We sent isolates with discordant intratypic differentiation results to the National Institute for Communicable Diseases, Johannesburg, South Africa, for entire VP1 sequencing according to WHO guidelines (*10*). To determine VP1 genetic diversity, we compared all complete VP1 sequences of type 2 cVDPV isolates from AFP patients and their contacts with the sequence of the Sabin-2 OPV reference strain (GenBank accession no. AY184220). We aligned sequences by using the ClustalW alignment program within the BioEdit Sequence Alignment Editor package version 7.0.9.0 (http://www.mbio.ncsu.edu/BioEdit/bioedit.html). The nearly complete genome of type 2 cVDPV isolate 2015-114-C6 was sequenced by deep sequencing. Random PCR and poliovirus-specific PCR products were generated and sequenced independently. We performed sequence amplification and analysis for the random approach according to Mee et al. (*13*) and for poliovirus-specific primers as described by Stern et al. (*14*). We used Geneious R10 software (https://www.geneious.com/) for deep-sequencing data analysis. The nucleotide sequence of type 2 cVDPV isolate 2015-114-C6 has been deposited in GenBank under accession no. MF346171.

### Phylogenetic and Recombination Analysis

For phylogenetic analyses, we used MEGA version 5.0 (http://www.megasoftware.net/). We determined sequence divergence by calculating mean pairwise distances within groups. To analyze its possible recombination structure, we compared the full-length genome sequence of isolate 2015-114-C6 with those of Sabin and WPV strains of the 3 serotypes and with prototype strains of 21 species of enteroviruses by using SimPlot version 3.5.1 (https://sray.med.som.jhmi.edu/SCRoftware/simplot/).

## Results

### Epidemiology

**Figure 1:**
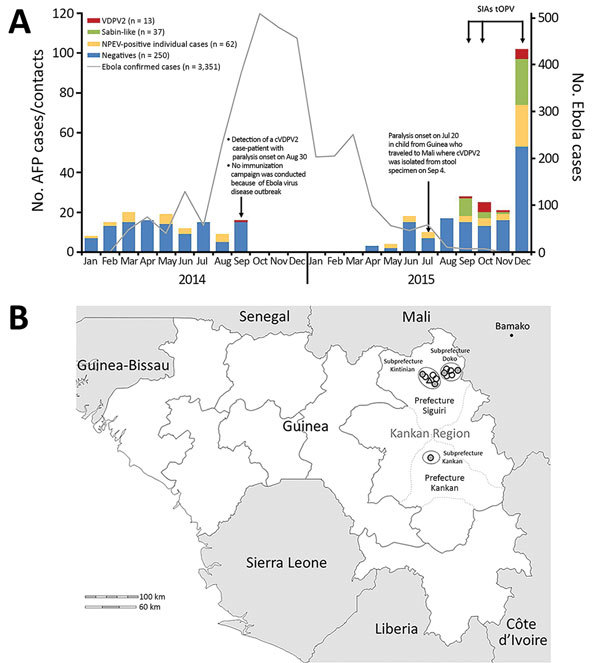
Epidemiologic context for emergence of vaccine-derived polioviruses during Ebola virus disease outbreak, Guinea, 2014–2015. A) Distribution of AFP cases (n = 132 in 2014; n = 113 in 2015) and contacts (n = 0 in 2014; n = 119 in 2015) for each month according to date of first fecal sample collection. Data for Ebola cases accessed at (*15*). B) Geographic distribution of case-patients (n = 6) and contacts (n = 7) with laboratory-confirmed VDPV2 infection. Outer circles indicate subprefectures; gray circles represent case-patients with paralysis onset in 2015; white circles represent laboratory-confirmed contacts; triangle represents case-patient with paralysis onset in 2014. AFP, acute flaccid paralysis; NPEV, nonpolio enterovirus; OPV, oral polio vaccine; tOPV, trivalent OPV; SIAs, supplementary polio immunization activities; VDPV2, type 2 vaccine-derived poliovirus.

During December 2013–May 2016, a total of 3,351 laboratory-confirmed cases of EVD occurred in Guinea, resulting in 2,083 deaths and reaching a peak of 509 confirmed cases in October 2014 (Figure 1, panel A). In September 2014, a case of laboratory-confirmed type 2 cVDPV infection was identified in Guinea. The case-patient (no. 14-120) was a 4-year-old boy in Siguiri Prefecture. On August 30, fever and bilateral paralysis of lower extremities developed; the patient was hospitalized 3 days later with AFP, and type 2 cVDPV strains were isolated from fecal samples on September 6 and 7. 

During October 2014–March 2015, collection of fecal samples from AFP patients in Guinea was interrupted because of the outbreak of EVD (Figure 1, panel A). On September 4, 2015, type 2 cVDPV was isolated from a fecal sample from a child from the Kankan region of Guinea; the child had become paralyzed on July 20 and was transported to Mali for treatment (*16*). Subsequently, type 2 cVDPV isolates were recovered from 5 AFP patients and 7 healthy contacts; dates of AFP onset were September–December 2015 (Figure 1, panel A). The 7 healthy type 2 cVDPV–positive contacts were epidemiologically linked to 3 of the AFP case-patients. Most (12/13) poliovirus-positive case-patients were incompletely vaccinated children (Table 1). All 13 type 2 cVDPV strains were isolated from persons in the Kankan region in eastern Guinea, near the border with Mali (Figure 1, panel B); most (12/13) persons were from Siguiri Prefecture. During the first semester of 2015, the coverage of routine OPV3 vaccination in Siguiri Prefecture was 31%. In 2014, the official national OPV3 routine coverage in Guinea was 42%. 

**Table 1:** Clinical and virologic features for case-patients and contacts from whom laboratory-confirmed type 2 cVDPVs were isolated from fecal samples, Guinea, 2014–2015*

Name†	Subpref‡	Paralysis onset date	Date of first sample	Paralysis within 3 d	Asymm. paralysis	No. OPV doses	Date of last OPV	No. VP1 nt changes vs. Sabin-2	% Match Sabin-2	GenBank accesion no.
Case-patient no., age, y/sex										
14-120, 4/M	Kintinian	2014 Aug 30	2014 Sep 6	Yes	No	1	2013 Oct 25	12	98.67	MF322697
15-078, 3/F	Kankan	2015 Sep 7	2015 Sep 10	No	No	2	2015 Jun 5	22	97.56	MF322698
15-114, 3/M	Kintinian	2015 Sep 30	2015 Oct 9	Yes	No	1	2012 May 12	20	97.78	MF322699
15-115, 1/M	Doko	2015 Oct 2	2015 Oct 18	No	No	1	2015 Sep 18	24	97.34	MF322700
15-136, 4/M	Kintinian	2015 Oct 10	2015 Nov 10	Yes	No	2	2015 Oct 1	27	97	MF322701
15-170, 1/M	Doko	2015 Dec 14	2015 Dec 18	No	Yes	0	NR	23	97.45	MF322702
Contact no., age, y/sex										
15-114-C6, 1/F	Kintinian	NA	2015 Dec 25	NA	NA	3	2015 Dec 6	22	97.56	MF322703
15-115-C4, 1/F	Doko	NA	2015 Oct 22	NA	NA	2	2015 Sep 28	22	97.56	MF322704
15-115-C5, 3/F	Doko	NA	2015 Oct 22	NA	NA	2	2015 Oct 29	22	97.56	MF322705
15-115-C8, 4/M	Doko	NA	2015 O t 23	NA	NA	2	2015 Sep 29	20	97.78	MF322706
15-115-C10, 0.58/F	Doko	NA	2015 Dec 23	NA	NA	1	2015 Dec 6	23	97.45	MF322707
15-136-C1, 1/F	Kintinian	NA	2015 Dec 11	NA	NA	1	2015 Dec 9	24	97.34	MF322708
15-136-C2, 0.5/F	Kintinian	NA	2015 Dec 10	NA	NA	1	2015 Dec 9	25	97.23	MF322709

*AFP, acute flaccid paralysis; Asymm., asymmetrical; cVDPV, circulating vaccine-derived poliovirus; NA, not applicable; NR, vaccine not received; OPV, oral polio vaccine; subpref, subprefecture; VP, capsid viral protein.  †All case-patients had AFP and fever at onset of paralysis. No contact had AFP. ‡All case-patients and contacts were from Siguiri Prefecture, except 15-078 (Doko Prefecture).

Households of the 5 children from whom laboratory-confirmed type 2 cVDPV was isolated (3 case-patients and 2 contacts) were investigated. All used man-made wells as the primary source of water and reported sharing a latrine with neighbors. None of the 5 households had been visited by children with AFP in the 3 months before paralysis onset. Parents all panned or mined for gold for a living and knew about polio but took no control measures to prevent infection and transmission. They all said they understood the value of vaccination and confirmed their commitment to vaccination activities.

We identified 59 potential contacts. All were incompletely vaccinated children. Of these 59 families, most (38 [64.4%]) were reluctant to allow fecal sample collection. 

In the 5 health centers investigated, we found nonfunctional refrigeration equipment and insufficient kits for sample collection and transportation. Frequent lack of polio vaccine stocks was also reported.

### Public Health Response and Vaccination Coverage

Since the first detection of a case of type 2 cVDPV infection in 2015 in Guinea, during 2015 and 2016, the Guinea Department of Health has conducted nationwide national immunization days (NIDs) and districtwide subnational immunization days (SNIDs) (Table 2). Assessments of vaccination coverage during the 3 campaigns in 2015 were conducted in Siguiri Prefecture. A total of 1,997 children were investigated (431 in SNIDs 1, 639 in SNIDs 2, and 927 in NIDs 1). The survey found that 181 (9%) of the 1,997 children remained unvaccinated. The most frequently reported reasons for not receiving OPV were absence at the time of vaccination (62.5%) and vaccination refusal (16.7%). The estimated OPV3 coverage in Siguiri Prefecture after the 3 SIAs of 2015 was 94%. Guinea’s official national OPV3 routine coverage for 2015 was 87%. Moreover, in late 2015 and in 2016 during SIAs, AFP surveillance activities were strengthened throughout the country. During 2016, a total of 2,585 fecal specimens from 1,298 children with AFP and 995 specimens from their 507 contacts were collected throughout Guinea. All specimens from 2016 were confirmed negative for VDPVs. Table 3 describes the evolution of the most representative AFP surveillance performance indicators in Guinea before, during, and after the polio outbreak.

**Table 2:** Supplementary polio immunization activities, Guinea, 2015–2016*

Dates	Type	Regions	Target population	No. vaccinated children	Vaccination coverage, %	Vaccine type
2015						
Sep 16–19	SNID	Faranah, Kankan, Nzérékoré, Labé	1,142,259	1,175,963	102.95	tOPV
Sep 28–Oct 1	SNID	Faranah, Kankan, Nzérékoré, Labé	1,175,963	1,224,364	104.12	tOPV
Dec 5–8	NID	Nationwide	2,523,431	2,497,033	98.95	tOPV
2016						
Jan 28–31	NID	Nationwide	2,611,902	2,738,818	104.86	tOPV
Mar 3–6	NID	Nationwide	2,611,902	2,883,669	110.40	tOPV
Apr 7–10	NID	Nationwide	2,880,679	3,066,638	106.46	tOPV
Apr 25–28	SNID	Faranah, Kankan	787,399	908,092	115.33	tOPV
Oct 6–9	NID	Nationwide	3,187,032	3,330,472	105.00	bOPV
Dec 2–5	NID	Nationwide	3,348,132	3,706,752	110.70	bOPV

*Vaccination coverage rates >100% result from vaccination of children who are not part of the initial target because of the movement of the population. bOPV, bivalent oral polio vaccine; NID, national immunization days; SNID, subnational immunization days; tOPV, trivalent oral polio vaccine.

**Table 3:** AFP surveillance quality indicators in Guinea, 2012–2016*

Indicator	Target	2012	2013	2014	2015	2016
AFP cases reported, no.	NA	186	221	132	113	1,298
AFP cases with 2 fecal specimens collected within 14 d of onset of paralysis, %	80	92.4	92.3	95.7	71.7	89.6
Fecal specimens arriving at national level within 3 d of being sent, %	80	83.4	87.9	87.8	79	58.8
Fecal specimens arriving at laboratory in good condition, %	90	63.7	54.6	86.4	94.3	94.2
Fecal specimens for which laboratory results were sent within 14 d of receipt at lab, %	80	100	100	99.2	93.1	62.5
Fecal specimens from which nonpolio enterovirus was isolated, %	10	12.8	8.5	12.4	7.5	16.1
VDPVs, no.	NA	0	0	1	12	0
Sabin virus, no.	NA	12	29	1	37	182

*AFP, acute flaccid paralysis; NA, not applicable; VDPV, vaccine-derived poliovirus.

### Isolates, Molecular Types, and VP1 Sequences

All 13 VDPV isolates showed discordant intratypic differentiation results and were further characterized by sequencing the VP1 capsid coding region (Table 4). All isolates diverged >0.6% nt from the type 2 OPV strain, which confirmed their classification as type 2 VDPVs (Table 1). All VDPV study isolates shared 8 nt substitutions, suggesting that all were derived from a common infection. The key determinant of Sabin-2 attenuated phenotype at nt 2909 of VP1 (*17*) reverted in all cVDPV strains (U_2909_→C resulting in an Ile_143_→Thr substitution) (Table 5).

**Table 4:** Frequency of samples, AFP cases, contacts, cVDPVs isolated, NPEV cases, and Sabin-like poliovirus cases in Guinea, 2012–2016*

Year	No. fecal samples received	No. AFP cases	No. contacts	No. cVDPVs isolated from case-patients	No. cVDPVs isolated from contacts	No. NPEV-positive cases	No. Sabin-like polioviruses
2012	366	185	0	0	0	23	12
2013	446	223	0	0	0	21	29
2014	258	132	0	1	0	22	1
2015	453	113	119	5	7	40	37
2016	3,580	1,298	507	0	0	363	182
*AFP, acute flaccid paralysis; cVDPV, circulating vaccine-derived poliovirus; NPEV, nonpolio enterovirus.							

**Table 5:** Nucleotide and amino acid substitutions in the vaccine part of the genome of vaccine-derived polioviruses, Guinea, 2014–2015*

Genomic region, nt or aa position	Sabin-2	15-114-C6	14-120	15-078	15-114	15-115	15-136	15-170	15-115-C4	15-115-C5	15-115-C8	15-115-C10	15-136-C1	15-136-C2	Phenotype
5′-UTR															
187	A	G													
202	C	T													
216	T	C													
290	G	A													
398	T	C													
438	A	G													
** 481**	**A**	**G**													Attenuation
653	G	C													
654	G	A													
655	T	C													
696	T	C													
710	A	G													
713	C	T													
718	A	G													
725	G	A													
736	A	G													
VP4															
* 47*	Ala	Thr													
VP3															
* 73*	Ser	Asn													NAg site 3
* 75*	Thr	Ala													NAg site 3
* 78*	Ser	Thr													NAg site 3
VP1															
* 4*	Asp	–	–	Asn	–	–	Ser	–	–	Gly	–	–	Gly	Gly	
* 10*	Val	Ile	Ile	Ile	Ile	Ile	Ile	Ile	Ile	Ile	Ile	Ile	Ile	Ile	
* 19*	Val	Ala	–	–	–	Ala	Ala	–	–	–	–	–	Ala	Ala	
* 23*	Ser	Pro	Pro	Pro	Pro	Pro	Pro	Pro	Pro	Pro	Pro	Pro	Pro	Pro	
* 24*	Thr	Ala	–	–	–	–	–	–	–	–	–	–	–	–	
* 26*	Ser	–	–	Gly	–	–	–	–	–	–	–	–	–	–	
* 30*	Thr	Ile	Ile	–	–	–	Ile	–	–	–	–	–	Ile	Ile	
* 31*	Lys	–	–	–	Arg	Arg	–	Arg	Arg	Arg	Arg	Arg	–	–	
* 43*	Ala	–	–	–	–	–	–	–	–	–	–	Val	–	–	
* 103*	Arg	Lys	–	–	–	–	–	–	–	–	–	–	–	Lys	NAg site 1
*** 143***	**Ile**	**Thr**	**Thr**	**Thr**	**Thr**	**Thr**	**Thr**	**Thr**	**Thr**	**Thr**	**Thr**	**Thr**	**Thr**	**Thr**	Attenuation
* 171*	Asn	–	–	–	–	–	Asp	–	–	–	–	–	Asp	Asp	NAg site 1

*In the 5′-UTR, all nucleotide positions differentiating VDPV 15-114-C6 from Sabin-2 are shown. In the capsid proteins region (VP4–VP1), only amino acid changes are shown. In the VP4-VP3 capsid protein regions, all amino acid positions differentiating VDPV 15-114-C6 from Sabin-2 are shown. For the VP1 capsid protein region, all amino acid positions differentiating all VDPVs from Guinea isolated in this study are shown. NAg, neutralizing antigen; UTR, untranslated region; VDPV, vaccine-derived poliovirus; VP, viral capsid protein. Boldface indicates relevant nucleotide or amino acid sites in the Sabin-2 genome described as being involved in Sabin-2 attenuation (*16*) or those predicted NAg sites important for immune recognition (*18*,*19*); italics indicate amino acid positions; – indicates no amino acid change; blank cells indicate no sequencing data.

### Phylogeny

**Figure 2:**
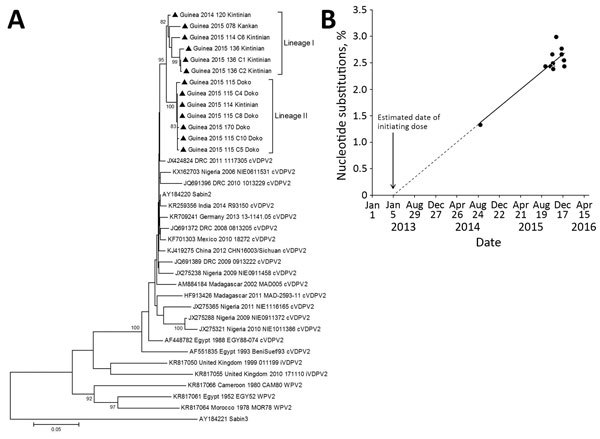
VP1 sequence analysis of cVDPV2 isolated from case-patients (n = 6) and contacts (n = 7) in Guinea, 2014–2015. A) Phylogenetic tree inferred with the complete VP1 region nucleotide sequences (903 bp). Our data were compared with a representative global set of 23 isolates representing type 2 VDPV strains from immunodeficient persons, Sabin-2 strain, cVDPV2s, and wild-type polioviruses identified by GenBank search. The Sabin-3 poliovirus sequence was introduced as an outgroup. The neighbor-joining tree was constructed by using MEGA 5.0 (http://www.megasoftware.net/). Distances were computed by using the Kimura 2-parameter model after excluding positions containing gaps and missing data from the alignments. The robustness of the nodes was tested by 1,000 bootstrap replications. Bootstrap support values >80 are shown in nodes. Triangles indicate the strains from this study. Study strains are indicated by the country, year of isolation, laboratory code, and subprefecture from which isolated. GenBank accession numbers for published sequences are shown in the tree. B) Estimation of the date of initial vaccination with Sabin-2, showing the proportion of VP1 nucleotide changes in the 13 cVDPVs isolates from Guinea with respect to the Sabin-2 reference vaccine strain (AY184220). The data were adjusted to a linear function for the accumulation of nucleotide substitutions (y = 0.0028*x + 1.372; R^2^ = 0.80). Date of initial OPV infection was calculated by extrapolating the line for the evolution rate of nucleotide substitutions backward to 0% substitutions. Scale bar represents nucleotide substitutions per site. cVDPV2, type-2 circulating vaccine-derived poliovirus; OPV, oral polio vaccine; VP, viral capsid protein.

All 13 type 2 cVDPV strains from Guinea clustered in a monophyletic group supported by a high (96%) bootstrap value (Figure 2, panel A). The close genetic relationship between the type 2 cVDPV strains from Guinea (97.8%–98.4% sequence similarity for nucleotides and 98.7%–99.3% for amino acids) supported the conclusion that all had a common precursor. The VP1 sequences segregated in 2 genetic lineages, which we designated I and II (with bootstrap values of 82% and 100%, respectively) and which probably correspond to different chains of transmission (Figure 2, panel A). A notable observation was the geographic segregation of type 2 cVDPV strains corresponding with the 2 observed genetic lineages. Strains isolated in the Kintinian subprefecture clustered in lineage I; those isolated in the Doko subprefecture clustered in lineage II (Figure 2, panel A). Estimates of average evolutionary divergence between study VDPV sequence pairs and the Sabin-2 OPV strain are shown in Table 6.

**Table 6:** Estimates of average evolutionary divergence over type-2 vaccine-derived poliovirus sequence pairs and the Sabin-2 oral poliovirus strain*

Location	nt p-distance value	SE	aa p-distance value	SE
Within lineage 1	0.017	± 0.003	0.009	± 0.004
Within lineage II	0.002	± 0.001	0.003	± 0.002
Between lineages I and II	0.028	± 0.004	0.013	± 0.005
Between lineage I – Sabin 2	0.025	± 0.005	0.021	± 0.007
Between lineage II – Sabin 2	0.024	± 0.005	0.015	± 0.006

*No. base substitutions/site or amino acid (aa) differences from averaging over all sequence pairs within or between each lineage are shown. Analyses were conducted by using the Kimura 2-parameter model and the pairwise distance at the nucleotide (nt) and aa levels, respectively. All positions containing gaps and missing data were eliminated. SES were obtained by a bootstrap procedure (1,000 replicates).

### Estimated Time of OPV Dose Initiation

We estimated the time of OPV dose initiation from the VP1 sequence divergence from Sabin-2 shown by the cVDPV isolates (Figure 2, panel B) and extrapolated the regression line for the evolution rate of nucleotide substitutions back to 0 in the Sabin-2 VP1. This date was estimated to be April 27, 2013 (95% CI February 10, 2012–December 7, 2013). When estimates were obtained for all VP1 sequences individually, with an assumed VP1 nucleotide substitution rate of 1% (*20*), the average date for initial OPV infection was April 20, 2013 (range November 13, 2012–July 18, 2013), remarkably similar to the above date estimated by linear regression. This finding suggests that all cVDPV isolates were derived from a single infection event.

### Whole-Genome Sequence of an Outbreak Type 2 cVDPV Strain

**Figure 3:**
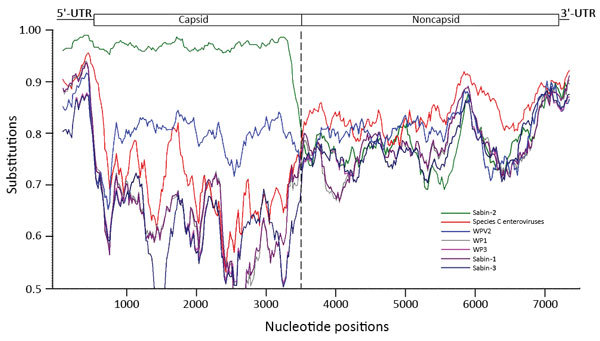
Whole-genome sequence analysis, showing similarity between cVDPV2 isolate 2015-114-C6 (query), 21 prototypes of human enterovirus species C, 3 WPVs, and 3 Sabin poliovirus strains. Approximate nucleotide positions in the poliovirus genome are indicated. The enterovirus genetic map is shown at top. Analyses were calculated by using SimPlot version 3.5 (https://sray.med.som.jhmi.edu/SCRoftware/simplot/). Similarity was calculated in each window of 300 bp by using the Kimura 2-parameter method with a transition:transversion ratio of 2. The window was advanced along the genome alignment in 20-bp increments with 1,000 resamplings. GenBank accession numbers for viruses tested: poliovirus type 1 (V01149), poliovirus type 2 (AY238473 and M12197), poliovirus type 3 (K01392); Sabin-1 (AY184219), Sabin-2 (AY184220), Sabin-3 (AY184221); coxsackie virus 1 (AF499635), coxsackie virus 11 (AF499636), coxsackie virus 13 (AF465511), coxsackie virus 17 (AF499639), coxsackie virus 19 (AF499641), coxsackie virus 20 (AF499642, EF015012, and EF015019), coxsackie virus 21 (AF465515 and D00538), coxsackie virus 22 (AF499643), coxsackie virus 24 (EF026081); enterovirus C96 (HQ415759), enterovirus C99 (EF555644), enterovirus C102 (EF555645), enterovirus C104 (JX982259), enterovirus C105 (KM880098), enterovirus C109 (GQ865517), enterovirus C117 (JX262382), enterovirus C116 (JX514942), and enterovirus C118 (JX961709). cVDPV2, type 2 circulating vaccine-derived poliovirus; UTR, untranslated region; WPV, wild-type poliovirus.

We subsequently obtained an almost-complete genomic sequence of type 2 VDPV isolate 2015-114-C6, from nt 33 to nt 7434 (Sabin-2 numbering) by deep sequencing and compared the sequence to that of the Sabin-2 vaccine reference strain. Consensus sequences obtained from both random and poliovirus-specific PCR products were identical and 100% similar to the original VP1 sequence determined by Sanger sequencing. Strain 2015-114-C6 contained an A-to-G nt substitution at 481 in the 5′-untranslated region (UTR), which represents the reversion of a major attenuation determinant of Sabin-2. This isolate differed from Sabin-2 at 807 nt and 65 aa substitutions in the open reading frame. Among all 65 aa changes, 30 represented reversions to amino acid residues found in MEF-1, a laboratory reference type-2 WPV strain. The outbreak isolate was a vaccine/nonvaccine recombinant; although 5′-UTR and P1 capsid genomic sequences were homologous to those of Sabin-2 (2.9% nt divergence), the noncapsid region (P2 + P3) and 3′-UTR sequences were dissimilar (18.2% nt divergence). The substitutions in the vaccine part of the VDPV genome are shown in Table 5. We estimated the crossover recombination point to be somewhere between nt 3262 and 3443. Similarity plot analysis revealed that nucleotide sequences in the 3′ half of the genome were dissimilar to those of other WPVs, vaccine poliovirus strains, or nonpolio species C enterovirus isolates (Figure 3). No isolates with sequence identity >84% to isolate 2015-114-C6 in the 3′ half of the genome were found in public sequence databases. We looked for circulating enteroviruses in samples from AFP cases that occurred in Guinea during 2013–2015. We found no enteroviruses of species C or D species (data not shown).

## Discussion

We isolated and characterized type 2 cVDPV strains during the 2014–2015 outbreak of EVD in Guinea. The origin and spread of the cVDPVs probably resulted from a combination of factors known to be associated with increased risk for cVDPV emergence and spread: insufficient population immunity against poliovirus and lack of high-quality and sensitive AFP surveillance (*3*). The low population immunity against poliovirus in Guinea resulted from low rates of routine OPV coverage and absence of indigenous circulation of the corresponding WPV serotype. According to administrative data, nationwide routine OPV3 coverage for 2014 was low (42%). During the first half of 2015, just before detection of the polio outbreak, routine OPV3 coverage in the affected prefecture of Siguiri was even lower (31%). An additional risk factor was poor household sanitation conditions linked to nonhygienic fecal disposal as revealed by our field investigations.

The concurrent EVD outbreak brought several challenges for poliovirus surveillance, vaccination, and diagnosis activities because most public health resources were used to detect and control EVD cases. First, AFP surveillance activities were interrupted from October 2014 through March 2015, just after detection of the first type 2 cVDPV case, which must have favored the undetectable spread of cVDPVs. Second, SIAs were cancelled and the quality of routine OPV vaccination services declined (*21*). Because the type 2 cVDPV detected in 2014 might represent hundreds to thousands of asymptomatic cVDPV infections (*7*), the fact that no follow-up SIAs were conducted might have fueled further spread. Similarly, emergence of other vaccine-preventable diseases, in particular measles, resulting from disruption of vaccination campaigns during the EVD outbreak have also been reported (*21*,*22*). Third, the EVD epidemic affected the processing of samples for polio diagnosis in the laboratory because of increased biosafety requirements. Samples were first tested for Ebola virus in a mobile laboratory in Conakry and then sent in batches of hundreds to the WHO Polio Reference Laboratory in Dakar. The shipping and processing of samples at 2 laboratories meant an exceptional workload in both laboratories and a shortage of supplies for diagnosis. These challenges for polio surveillance, vaccination, and diagnosis argue for the development of contingency plans during complex emergencies to ensure completion and maintenance of global polio eradication.

Vaccination activities conducted in 2015 and 2016 most likely contributed to the disruption of VDPV transmission because, despite an intensive search, no polio cases were detected in Guinea after December 2015. However, the country remains vulnerable to polio importation because gaps in AFP surveillance continue to occur in some areas and routine OPV coverage recorded for 2015 (87%) and 2016 (86%) remained suboptimal.

The area where the outbreak occurred is a gold mining region. Field investigations revealed that all parents of the investigated case-patients were gold miners or panners. There is a probable epidemiologic link between cases because of the frequent migratory movements in these gold mining areas, especially in the subprefectures of Kintinian and Doko. Gold panning sites are areas of high risk because of precarious living conditions; attention should be focused on strengthening and prioritizing vaccination efforts (routine and SIAs) in these specific populations. Field investigations also revealed hesitancy by some families to vaccinate and reluctance of some contacts’ families to collect fecal samples from their children. These findings call for social mobilization efforts to increase vaccination trust and polio surveillance adherence in these communities.

We found that the clinical characteristics of VDPV infections were similar to those associated with WPV infection. Available clinical data showed that all cVDPV case-patients had residual paralysis after 60 days, typical of poliomyelitis.

Genetic analysis of cVDPVs isolates was consistent with the 2 most serious biological properties of cVDPVs: capacity for sustained person-to-person transmission and capacity to cause paralytic disease in humans. Effectively, the findings that the VDPV isolates had high genetic divergence from the Sabin-2 vaccine strain in the VP1 coding region (1.3%–3% nt differences) and a vaccine/nonvaccine recombinant genome are indications of sustained circulation of VDPVs. Moreover, our finding that the 2 key genetic determinants of the attenuated phenotype of the Sabin-2 strain (A481G in the 5′-UTR and I143T in the VP1 [*17*,*23*]) have reverted points to the cause of the paralysis. Recombination of vaccine strains with other species C enteroviruses and replacement of the 2 key attenuating substitutions of the Sabin-2 strain are features frequently seen among type 2 VDPVs reported in previous outbreaks (*18*,*24*–*27*).

Phylogenetic analysis of VP1 sequences suggests that all type-2 cVDPV strains diverged from a common OPV precursor and circulated along 2 chains of transmission that gave rise to 2 descending lineages. Each lineage was restricted to a limited geographic region, Kintinian or Doko. Our results suggest that viruses from lineage II, found in Doko, might have been derived from strain 2015-114 (or a closely related sibling), which was the only strain from Kintinian classified in lineage II. Given the low nationwide rate of routine vaccination coverage, it is surprising that the outbreak was geographically confined to the region of Kankan. This localization could be the result of limited movement of populations. Without a developed long-range transportation network, individual geographic regions tend to be isolated, limiting the movement of human populations and thus restricting widespread virus transmission (*28*). Alternatively, the absence of widespread circulation may also indicate that VDPV strains are less transmissible than WPV.

In conclusion, epidemiologic and molecular data show that the outbreak VDPV strain circulated since 2013 in Guinea in an area of low vaccination coverage and substantial gaps in population immunity at a critical time in the polio endgame. This finding reiterates the need to enhance vaccination activities focused on risk groups to maintain high OPV coverage and strengthen routine vaccination and sensitive AFP surveillance systems to ensure the quick detection of cVDPV emergence. Our study shows how diversion of public health resources to the EVD outbreak in Guinea may have contributed to the spread of neurovirulent VDPVs. This finding argues strongly for enhancement of polio surveillance and vaccination efforts in other EVD-affected countries, such as neighboring Liberia and Sierra Leone, where vaccination systems have been weakened and where active AFP surveillance has only recently started (*29*). The fact that this outbreak occurred just before the withdrawal of type 2 from OPV adds significance because there was a particular urgency for stopping type 2 VDPV transmission before the withdrawal target date. In this context, as shown in our study, effective and sensitive surveillance for polio, as well as a good understanding of the extent and natural history of the outbreak, have proven critical for designing the best public health response.

## References

[1] Marx A, Glass JD, Sutter RW. Differential diagnosis of acute flaccid paralysis and its role in poliomyelitis surveillance. Epidemiol Rev. 2000;22:298–316. 10.1093/oxfordjournals.epirev.a018041 11218380

[2] World Health Organization. Poliomyelitis [cited 2017 Jan 1]. http://www.who.int/mediacentre/factsheets/fs114/en/

[3] Kew OM, Sutter RW, de Gourville EM, Dowdle WR, Pallansch MA. Vaccine-derived polioviruses and the endgame strategy for global polio eradication. Annu Rev Microbiol. 2005;59:587–635. 10.1146/annurev.micro.58.030603.123625 16153180

[4] Kew O, Morris-Glasgow V, Landaverde M, Burns C, Shaw J, Garib Z, et al. Outbreak of poliomyelitis in Hispaniola associated with circulating type 1 vaccine-derived poliovirus. Science. 2002;296:356–9. 10.1126/science.1068284 11896235

[5] Jorba J, Diop OM, Iber J, Sutter RW, Wassilak SG, Burns CC. Update on vaccine-derived polioviruses—worldwide, January 2015–May 2016. MMWR Morb Mortal Wkly Rep. 2016;65:763–9. 10.15585/mmwr.mm6530a3 27491079

[6] Burns CC, Diop OM, Sutter RW, Kew OM. Vaccine-derived polioviruses. J Infect Dis. 2014;210(Suppl 1):S283–93. 10.1093/infdis/jiu295 25316847

[7] Diop OM, Burns CC, Sutter RW, Wassilak SG, Kew OM; Centers for Disease Control and Prevention (CDC). Update on vaccine-derived polioviruses—worldwide, January 2014–March 2015. MMWR Morb Mortal Wkly Rep. 2015;64:640–6. 26086635 PMC4584736

[8] World Health Organization. WHO-recommended standards for surveillance of selected vaccine-preventable diseases. February 2003 [cited 2017 Jan 1]. http://apps.who.int/iris/bitstream/10665/68334/1/WHO-V-B_03.01_eng.pdf?ua=1

[9] Diop OM, Burns CC, Wassilak SG, Kew OM; Centers for Disease Control and Prevention (CDC). Update on vaccine-derived polioviruses - worldwide, July 2012-December 2013. MMWR Morb Mortal Wkly Rep. 2014;63:242–8. 24647401 PMC4584635

[10] World Health Organization. Polio Laboratory Manual. 4th edition. 2004 [cited 2017 Jan 1]. http://apps.who.int/iris/bitstream/10665/68762/1/WHO_IVB_04.10.pdf

[11] Kilpatrick DR, Ching K, Iber J, Chen Q, Yang SJ, De L, et al. Identification of vaccine-derived polioviruses using dual-stage real-time RT-PCR. J Virol Methods. 2014;197:25–8. 10.1016/j.jviromet.2013.11.017 24321704 PMC8215842

[12] Fernandez-Garcia MD, Kebe O, Fall AD, Ndiaye K. Identification and molecular characterization of non-polio enteroviruses from children with acute flaccid paralysis in West Africa, 2013-2014. Sci Rep. 2017;7:3808. 10.1038/s41598-017-03835-1 28630462 PMC5476622

[13] Mee ET, Minor PD, Martin J. High resolution identity testing of inactivated poliovirus vaccines. Vaccine. 2015;33:3533–41. 10.1016/j.vaccine.2015.05.052 26049003 PMC4504004

[14] Stern A, Yeh MT, Zinger T, Smith M, Wright C, Ling G, et al. The evolutionary pathway to virulence of an RNA virus. Cell. 2017;169:35–46 e19. 10.1016/j.cell.2017.03.013 PMC5787669 28340348

[15] World Health Organization. Ebola data and statistics. Data published on 11 May 2016. Data on new cases per epi week for Guinea [cited 2017 Jan 1]. http://apps.who.int/gho/data/view.ebola-sitrep.ebola-country-GIN-20160511-data?lang=en

[16] Morales M, Nnadi CD, Tangermann RH, Wassilak SG. Notes from the field: circulating vaccine-derived poliovirus outbreaks—five countries, 2014–2015. MMWR Morb Mortal Wkly Rep. 2016;65:128–9. 10.15585/mmwr.mm6505a5 26866942

[17] Macadam AJ, Pollard SR, Ferguson G, Skuce R, Wood D, Almond JW, et al. Genetic basis of attenuation of the Sabin type 2 vaccine strain of poliovirus in primates. Virology. 1993;192:18–26. 10.1006/viro.1993.1003 8390752

[18] Adu F, Iber J, Bukbuk D, Gumede N, Yang SJ, Jorba J, et al. Isolation of recombinant type 2 vaccine-derived poliovirus (VDPV) from a Nigerian child. Virus Res. 2007;127:17–25. 10.1016/j.virusres.2007.03.009 17449127

[19] Shulman LM, Manor Y, Handsher R, Delpeyroux F, McDonough MJ, Halmut T, et al. Molecular and antigenic characterization of a highly evolved derivative of the type 2 oral poliovaccine strain isolated from sewage in Israel. J Clin Microbiol. 2000;38:3729–34. 11015392 10.1128/jcm.38.10.3729-3734.2000 PMC87465

[20] Jorba J, Campagnoli R, De L, Kew O. Calibration of multiple poliovirus molecular clocks covering an extended evolutionary range. J Virol. 2008;82:4429–40. 10.1128/JVI.02354-07 18287242 PMC2293050

[21] Delamou A, El Ayadi AM, Sidibe S, Delvaux T, Camara BS, Sandouno SD, et al. Effect of Ebola virus disease on maternal and child health services in Guinea: a retrospective observational cohort study. Lancet Glob Health. 2017;5:e448–57. 10.1016/S2214-109X(17)30078-5 28237252 PMC6530984

[22] Colavita F, Biava M, Castilletti C, Quartu S, Vairo F, Caglioti C, et al.; Lazzaro Spallanzani Institute for Research and Health Care Ebola Virus Disease Sierra Leone Study Group. Measles cases during Ebola outbreak, West Africa, 2013–2106. Emerg Infect Dis. 2017;23:1035–7. 10.3201/eid2306.161682 28518027 PMC5443435

[23] Ren RB, Moss EG, Racaniello VR. Identification of two determinants that attenuate vaccine-related type 2 poliovirus. J Virol. 1991;65:1377–82. 1847458 10.1128/jvi.65.3.1377-1382.1991 PMC239915

[24] Yan D, Zhang Y, Zhu S, Chen N, Li X, Wang D, et al. Limited and localized outbreak of newly emergent type 2 vaccine-derived poliovirus in Sichuan, China. Clin Vaccine Immunol. 2014;21:1012–8. 10.1128/CVI.00196-14 24850620 PMC4097442

[25] Rakoto-Andrianarivelo M, Guillot S, Iber J, Balanant J, Blondel B, Riquet F, et al. Co-circulation and evolution of polioviruses and species C enteroviruses in a district of Madagascar. PLoS Pathog. 2007;3:e191. 10.1371/journal.ppat.0030191 18085822 PMC2134956

[26] Rakoto-Andrianarivelo M, Gumede N, Jegouic S, Balanant J, Andriamamonjy SN, Rabemanantsoa S, et al. Reemergence of recombinant vaccine-derived poliovirus outbreak in Madagascar. J Infect Dis. 2008;197:1427–35. 10.1086/587694 18419577

[27] Burns CC, Shaw J, Jorba J, Bukbuk D, Adu F, Gumede N, et al. Multiple independent emergences of type 2 vaccine-derived polioviruses during a large outbreak in northern Nigeria. J Virol. 2013;87:4907–22. 10.1128/JVI.02954-12 23408630 PMC3624331

[28] Gumede N, Lentsoane O, Burns CC, Pallansch M, de Gourville E, Yogolelo R, et al. Emergence of vaccine-derived polioviruses, Democratic Republic of Congo, 2004-2011. Emerg Infect Dis. 2013;19:1583–9. 10.3201/eid1910.130028 24047933 PMC3810735

[29] World Health Organization. Statement of the 11th IHR Emergency Committee regarding the international spread of poliovirus. November 11, 2016 [cited 2017 Jan 1]. http://www.who.int/mediacentre/news/statements/2016/11th-ihr-polio/en/

